# Predictors of HBsAg seroclearance in patients with chronic HBV infection treated with pegylated interferon-α: a systematic review and meta-analysis

**DOI:** 10.1007/s12072-024-10648-8

**Published:** 2024-03-09

**Authors:** Shaowen Jiang, Simin Guo, Yan Huang, Yalin Yin, Jingwen Feng, Huijuan Zhou, Qing Guo, Weijing Wang, Haiguang Xin, Qing Xie

**Affiliations:** 1 Department of Infectious Diseases, Ruijin Hospital, Shanghai Jiao Tong University School of Medicine, Shanghai, China; 2 School of Life Sciences, Xiamen University, Xiamen, China; 3 School of Pharmacy, Chengdu University of Traditional Chinese Medicine, Chengdu, China; 4 Department of Infectious Diseases, Ruijin Hospital North, Shanghai Jiao Tong University School of Medicine, Shanghai, China

**Keywords:** Chronic HBV infection, Pegylated interferon-α, HBsAg seroclearance, Meta-analysis, Predictors

## Abstract

**Background and aims:**

The identification of reliable predictors for hepatitis B surface antigen (HBsAg) seroclearance remains controversial. We aimed to summarize potential predictors for HBsAg seroclearance by pegylated interferon-α (PegIFNα) in patients with chronic HBV infection.

**Methods:**

A systematic search of the Cochrane Library, Embase, PubMed, and Web of Science databases was conducted from their inception to 28 September 2022. Meta-analyses were performed following the PRISMA statement. Predictors of HBsAg seroclearance were evaluated based on baseline characteristics and on-treatment indicators.

**Results:**

This meta-analysis encompasses 27 studies, including a total of 7913 patients. The findings reveal several factors independently associated with HBsAg seroclearance induced by PegIFNα-based regimens. These factors include age (OR = 0.961), gender (male vs. female, OR = 0.537), genotype (A vs. B/D; OR = 7.472, OR = 10.738), treatment strategy (combination vs. monotherapy, OR = 2.126), baseline HBV DNA (OR = 0.414), baseline HBsAg (OR = 0.373), HBsAg levels at week 12 and 24 (OR = 0.384, OR = 0.294), HBsAg decline from baseline to week 12 and 24 (OR = 6.689, OR = 6.513), HBsAg decline from baseline ≥ 1 log_10_ IU/ml and ≥ 0.5 log_10_ IU/ml at week 12 (OR = 18.277; OR = 4.530), and ALT elevation at week 12 (OR = 3.622). Notably, subgroup analysis suggests no statistical association between HBsAg levels at week 12 and HBsAg seroclearance for treatment duration exceeding 48 weeks. The remaining results were consistent with the overall analysis.

**Conclusions:**

This is the first meta-analysis to identify predictors of HBsAg seroclearance with PegIFNα-based regimens, including baseline and on-treatment factors, which is valuable in developing a better integrated predictive model for HBsAg seroclearance to guide individualized treatment and achieve the highest cost-effectiveness of PegIFNα.

**Supplementary Information:**

The online version contains supplementary material available at 10.1007/s12072-024-10648-8.

## Introduction

Hepatitis B virus (HBV) infection is a significant global public health issue. According to the World Health Organization (WHO), approximately 296 million people suffer from chronic HBV infection, and there are 1.5 million new infections each year worldwide [1]. Chronic hepatitis B (CHB) can lead to liver fibrosis, cirrhosis, hepatocellular carcinoma, and premature death. Moreover, it is associated with an increased risk of several other types of cancers [2]. Without proper treatment, it is estimated that hepatitis B-related deaths will reach 1.14 million by 2035 [3]. In China, the number of individuals with chronic HBV infections is remarkably high, estimated around 80 million in 2022, making it the leading country in terms of hepatitis B surface antigen (HBsAg)-positive populations [4]. While China has made considerable progress in the prevention and treatment of hepatitis B, diagnosis and treatment concepts are evolving rapidly [5]. Despite these efforts, achieving the WHO's goal of "eliminating viral hepatitis as a public health hazard by 2030" remains a significant challenge [6].

The ideal treatment goal for CHB patients is achieving HBsAg seroclearance or seroconversion, considered as a functional cure, as endorsed by authoritative national and international guidelines [6–9]. However, the rate of spontaneous HBsAg seroclearance in adult CHB patients is only 1.17% per year [10], and even lower at 1% for children [11]. Current antiviral drugs for HBV mainly include interferon-α (containing both conventional interferon-α and pegylated interferon-α) and nucleos(t)ide analogues (NAs). The annual incidence of HBsAg seroclearance with NAs is a mere 0.22%, with a 10-year cumulative incidence of 2.11% [12]. In contrast, studies have shown that pegylated interferon-α (PegIFNα)-based therapy has significantly improved functional cure rates. For instance, PegIFNα-based regimens resulted in a high HBsAg seroclearance rate of 47% in inactive HBsAg carriers (IHCs), compared to only 1.54% in the control group (including NAs-treated or untreated patients) [13]. PegIFNα-based therapy has also shown promising results in patients with prior NAs experience [14, 15], those with low-level viremia [16], pediatric patients [17, 18], and postpartum individuals [19], achieving over 30% HBsAg seroclearance rates. Treatment-naive patients treated with PegIFNα for 48 weeks achieved approximately 10% HBsAg seroclearance rate [20]. Moreover, a meta-analysis of 49 studies indicated that interferon-α combined with NAs therapy significantly improved HBsAg seroclearance compared to NAs monotherapy, though no statistically significant difference was found compared to interferon-α monotherapy [21]. Despite these advancements, HBsAg seroclearance rates in treatment-naive patients remain unsatisfactory. Research on individualized treatment approaches has suggested that extending PegIFNα-based treatment or implementing intermittent treatment strategies may increase the likelihood of achieving HBsAg seroclearance [22, 23]. Long-term follow-up studies have demonstrated the durability of HBsAg seroclearance achieved through interferon-α treatment and a remarkably low risk of hepatocellular carcinoma post-seroclearance [24, 25]. Recent guideline recommends the addition of PegIFNα in selected populations to pursue functional cure [6].

Expanding the scope of antiviral therapy to benefit a broader range of patients with CHB and optimizing treatment to increase the functional cure rate and improve long-term prognosis are key areas of exploration [6–8]. Currently, several clinical trials have shown that certain baseline characteristics, on-treatment viral and immunological biomarkers, can serve as potential predictors of HBsAg seroclearance, offering valuable guidance for clinical decision-making and treatment strategies [26–29]. However, the relationship between these predictors and HBsAg seroclearance remains uncertain due to variations in patients' characteristics, treatment duration, and sample size among different studies [30–33]. Therefore, this systematic review and meta-analysis aim to comprehensively summarize the predictors of HBsAg seroclearance in patients with chronic HBV infection treated with PegIFNα, by assessing baseline characteristics and monitoring on-treatment indicators. The results of this study will facilitate the development of individualized antiviral treatment strategies in clinical practice, leading to an improvement in the functional cure rate and a reduction in the incidence of adverse outcomes related to chronic HBV infection.

## Materials and methods

The Preferred Reporting Items for Systematic Reviews and Meta-Analyses (PRISMA) guidelines were followed as a standard for conducting the meta-analysis. The protocol for this study has been registered in the PROSPERO registry (CRD42023418241).

### Literature search

Two researchers (Shaowen Jiang and Simin Guo) conducted a comprehensive search across multiple databases, including PubMed, EMBASE, the Cochrane Central Register of Controlled Trials, and Web of Science, using the PICOS standard as a guide. The search aimed to identify relevant articles published from the inception of the databases until 28 September 2022. Subject terms and free words such as "Hepatitis B, Chronic," "Hepatitis B Surface Antigens," "Seroconversion," and "Clinical Trial" were used in the search strategy (complete search strategy in Supplementary Table 1). After eliminating duplicates, Shaowen Jiang and Simin Guo meticulously screened each entry to ensure adherence to the inclusion criteria. In addition, a comprehensive literature search was conducted and kept update during the screening process, and the reference lists of retrieved original reports and review articles were scrutinized to identify any additional studies that had not been included.

### Inclusion and exclusion criteria

The following criteria were used for inclusion: (1) clinical trial studies published in English; (2) adult patients with chronic hepatitis B virus infection; (3) treated with PegIFNα once a week for ≥ 24 weeks, either in combination with or without NAs; (4) HBsAg seroclearance defined as HBsAg < 0.05 IU/mL with or without HBsAb (hepatitis B surface antibody) > 10 mIU/mL; (5) reported at least one predictor of HBsAg seroclearance following PegIFNα monotherapy or PegIFNα-based therapy; (6) multivariate Cox or logistic regression must be utilized in statistical analysis of predictor for HBsAg seroclearance by PegIFNα-containing therapy.

The following criteria were used for exclusion: (1) literature published in formats, such as reviews, patents, guidelines, chapters, case reports, conference abstracts, letters, or editorials; (2) studies based on small sample sizes (< 10 patients); (3) patients who were co-infected with hepatitis C, hepatitis D, HIV, etc.; (4) studies in which a large proportion (> 50%) of patients received NAs monotherapy treatment in the analyses of predictors for HBsAg seroclearance; and (5) articles containing insufficient data or obviously doubtful data. In cases where multiple studies included overlapping populations, only articles with relatively complete or extractable data were included.

### Data extraction

Two researchers (Yan Huang and Jingwen Feng), with extensive experience in the field of hepatitis B, independently screened the literature based on predetermined inclusion and exclusion criteria. They used a standardized data extraction form to extract information, cross-checked each case, and resolved any disagreements through discussion with a third researcher (Shaowen Jiang). The extracted data included: (1) study characteristics: name of first author, country, year of publication, type of study; (2) basic patient information: sample size, age, gender; (3) treatment modality: treatment regimen, duration of therapy, length of follow-up after discontinuation of PegIFNα; and (4) predictors of HBsAg seroclearance prioritized for evaluating outcomes at the end of follow-up (if reported); if not, end-of-treatment outcomes were assessed.

### Quality assessment

The Cochrane risk of bias was used to assess the quality of the randomized controlled trials (RCTs) [34], while the Newcastle–Ottawa Quality Assessment Scale (NOS) was used for cohort studies [35]. The MINORS score was used for single-arm studies [36]. Quality assessment was performed independently by two investigators, and discrepancies were resolved by a third investigator.

### Statistical analysis

The primary objective of this study is to extract effect estimates, such as hazard ratio (HR) or odds risk (OR), from multivariate Cox or logistic regression models, along with their corresponding 95% confidence intervals (CIs) for each risk factor related to the outcome of interest. For statistical analysis, predictors were considered only if relevant data from at least two studies were available. To quantify statistical heterogeneity, the *I*^*2*^ and Cochrane Q tests were employed, which assessed the proportion of variation between studies attributable to heterogeneity rather than chance. When *I*^*2*^ > 50% and *p* value < 0.05 indicated substantial heterogeneity, a random-effects model was utilized. Conversely, a fixed-effects model was used in the absence of significant heterogeneity, with sensitivity analysis to verify the results. Subgroup analyses were performed to evaluate the impact of region and treatment duration on the pooled estimates. Meta-regression analysis was used to obtain the source of high heterogeneity for findings on some factors reported in more than 10 articles. Publication bias in the literature was assessed using Egger and Begge tests, with a minimum of seven articles required for the analysis. If evidence of publication bias was detected, the Trim and Fill procedure was applied. Statistical significance was set at a *p* value of less than 0.05. All statistical analyses were conducted using Stata software version 15.0 (Stata Corp., College Station, TX, USA). Quality assessment of RCT studies was performed using Review Manager 5.3.

## Results

**Fig. 1:**
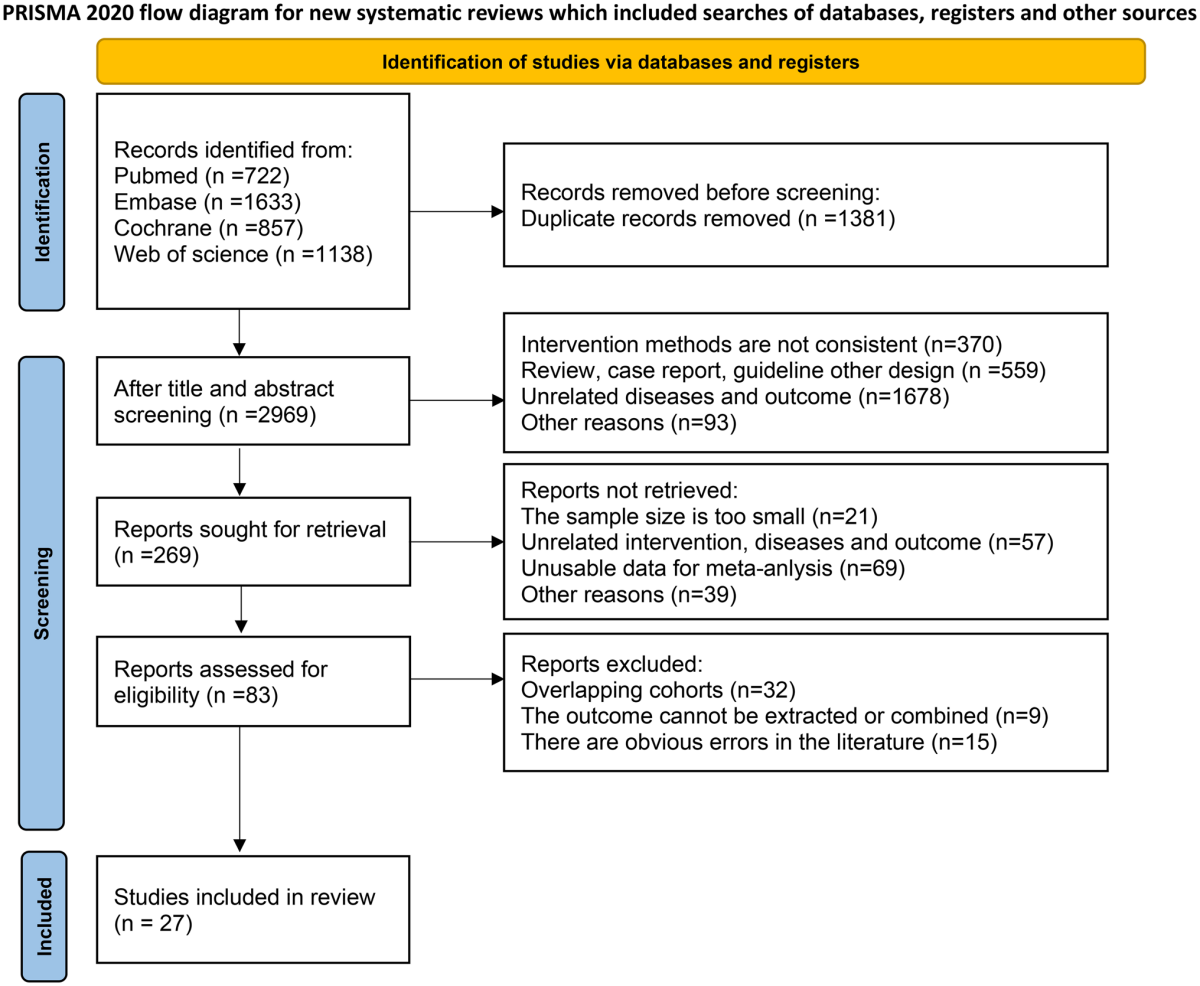
Flow diagram showing the selection of studies for the meta-analysis

Figure 1 illustrates the study selection process. Initially, 4350 publications were identified using the search terms. After screening titles and abstracts, 269 studies were considered potentially relevant and underwent a full review. Among these, 242 studies were excluded due to various reasons, including a lack of reporting on multivariate analysis predictors, duplicate patient data, or other issues. Eventually, 27 studies were included in the meta-analysis.

### Study characteristics

The analysis included a total of 27 studies conducted between 2009 and 2023, involving 7913 patients, of which 5706 (72.1%) were male. The studies exhibited substantial variations in sample size, ranging from 42 to 2579 participants, with most being conducted in China (22 studies), followed by 3 studies in the Netherlands, 1 in France, and 1 in Thailand. For study types, it comprised 13 multi-center studies and 14 single-center studies; 5 randomized controlled trials (RCTs), 9 cohort studies, and 13 single-arm trials. Among these, 17 (62.9%) studies were prospective, while the remaining 10 were retrospective. 7 studies were treatment-naive patients; 10 were NAs-treated patients; 10 included both treatment-naive and NAs-treated patients/ undisclosed. In addition, 4 studies included HBeAg (hepatitis B e antigen) positive patients, 14 included HBeAg negative patients, and 9 included both HBeAg positive and negative patients. Furthermore, 3 studies focused on IHCs, and one of these also included patients with chronic HBV infection. The treatment durations varied, with 48 weeks being the most common (17 studies), followed by 24–48 weeks in 2 studies, 48–96 weeks in 4 studies, 96 weeks in 3 studies, and over 96 weeks in one study. The follow-up period after discontinuation of PegIFNα treatment ranged from 0 to 360 weeks (Supplementary Table 2).

### Quality assessment

**Fig. 2:**
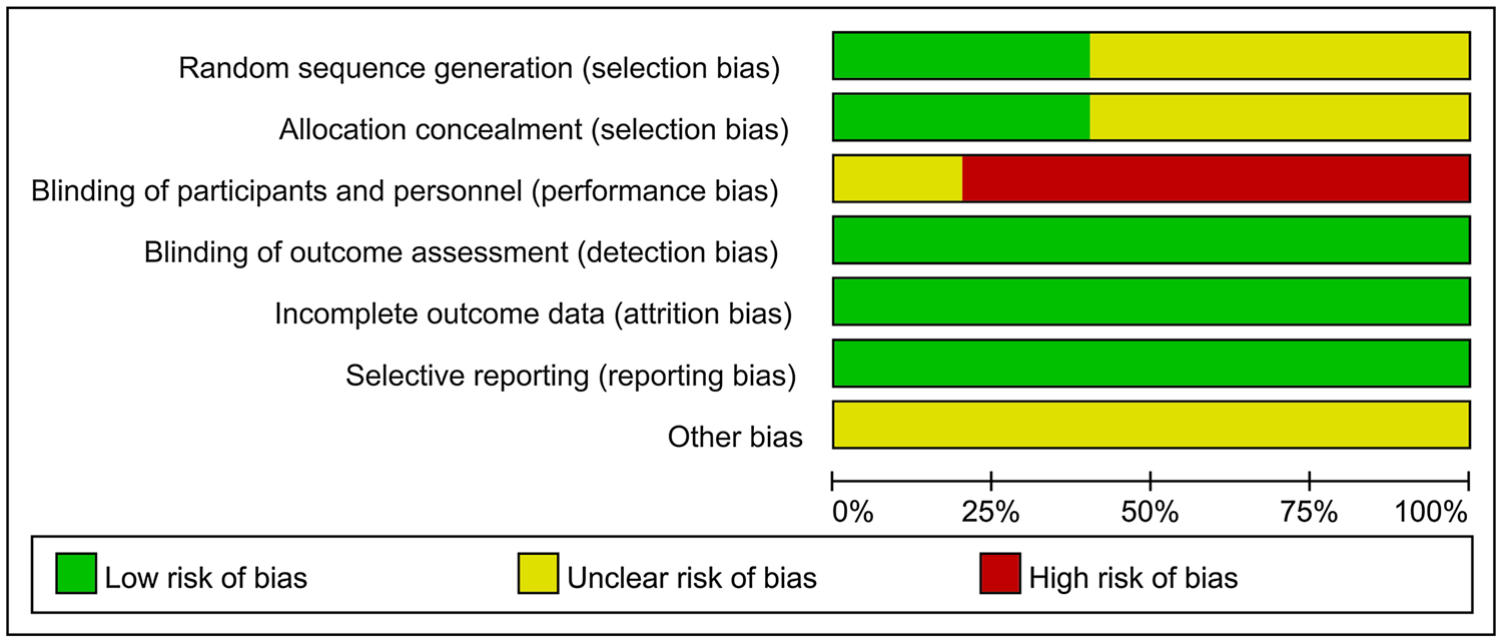
Quality assessment of randomized control trials

The RCTs demonstrated a low risk of test bias and missed visits, thanks to the use of quantitative laboratory indicators and a missed visit rate of less than 20%. However, selection bias remained unclear in three RCTs due to unreported randomization and allocation methods. Furthermore, implementation bias was high across four RCTs as they used open-label controlled trials (Fig. 2). Each of the nine cohort studies exhibited a low risk score ranging between 7 and 9 (Supplementary Table 3). Similarly, the 12 single-arm studies also demonstrated a low risk score, ranging from 12 to 15 (Supplementary Table 4).

### Correlation between baseline characteristics and HBsAg seroclearance

Among the 27 included studies, the correlation between age (in years), gender, genotype (A, B, D), treatment strategy (combination with NAs vs. monotherapy), baseline HBsAg (log_10_ IU/ml), HBeAg (Log S/CO), HBV DNA (log_10_ IU/mL), and HBsAg seroclearance was reported in 8, 2, 4, 2, 14, 2, and 4 studies, respectively.

Table 1 presents the combined estimates of baseline factors that were reported in two or more studies. Regarding patient characteristics, a younger age showed a positive correlation with HBsAg seroclearance (OR = 0.961, 95% CI 0.925–0.997, *I*^*2*^ = 61.6%). There was a significant difference in sample size between the two gender-related studies (2579 vs. 60), with males demonstrating greater difficulty in achieving HBsAg seroclearance compared to females (OR = 0.537, 95% CI 0.371–0.777, *I*^*2*^ = 0%). Concerning baseline virological indicators, patients with genotype A were more likely to achieve HBsAg seroclearance than those with genotype D (OR = 10.738, 95% CI 3.098–37.215, *I*^*2*^ = 0%), and genotype B (OR = 7.472, 95% CI 2.549–21.897, *I*^*2*^ = 0%). In addition, treatment with PegIFNα combined with NAs demonstrated a higher probability of achieving HBsAg seroclearance compared to PegIFNα monotherapy (OR = 2.126, 95% CI 1.059–4.269, *I*^*2*^ = 0%). Furthermore, lower baseline HBsAg (log_10_ IU/ml) and HBV DNA (log_10_ IU/mL) were significantly associated with HBsAg seroclearance (OR = 0.373, 95% CI 0.266–0.522, *I*^*2*^ = 56.1%; OR = 0.414, 95% CI 0.253–0.678, *I*^*2*^ = 16.9%). However, baseline HBeAg (log S/CO) did not show a statistically significant relationship with HBsAg seroclearance (OR = 0.877, 95% CI 0.556–1.384, *I*^*2*^ = 0%) (Supplementary Figs. 1–3).

**Table 1:** Baseline guided therapy (BGT) predictors associated with HBsAg seroclearance

Predictors	HBsAg seroclearance		
	No. study	OR (95% CI)	*p* value
Age (year)	8	0.961 (0.925, 0.997)	0.035*
PegIFNα treatment ≤ 48 weeks	6	0.978 (0.933, 1.026)	0.363
PegIFNα treatment > 48 weeks	2	0.917 (0.875, 0.961)	< 0.001*
Gender (male vs. female)	2	0.537 (0.371, 0.777)	0.001*
Genotype			
A versus D	2	10.738 (3.098, 37.215)	< 0.001*
A versus B	2	7.472 (2.549, 21.897)	< 0.001*
Combination with NAs (yes vs. no)	2	2.126 (1.059, 4.269)	0.034*
Baseline HBsAg (log_10_ IU/mL)	14	0.373 (0.266, 0.522)	< 0.001*
PegIFNα treatment ≤ 48 weeks	10	0.322 (0.205, 0.506)	< 0.001*
PegIFNα treatment > 48 weeks	4	0.462 (0.251, 0.850)	0.013*
Baseline HBeAg (log S/CO)	2	0.877 (0.556, 1.384)	0.574
Baseline HBV DNA (log _10_ IU/mL)	4	0.414 (0.253, 0.678)	< 0.001*
PegIFNα treatment ≤ 48 weeks	2	0.218(0.086, 0.555)	0.002*
PegIFNα treatment > 48 weeks	2	0.531 (0.297, 0.950)	0.033*

*Significantly associated with HBsAg seroclearance

### Correlation of on-treatment virological and biochemical factors with HBsAg seroclearance

Among the 27 studies, the relationship of HBsAg (log_10_ IU/mL) during treatment, HBsAg (IU/mL) at week 12, HBsAg (log_10_ IU/mL) decline from baseline to week 12 and 24, HBsAg decline from baseline to week 12 (≥ 1 vs. < 1 log_10_ IU/mL, ≥ 0.5 vs. < 0.5 log_10_ IU/mL), and ALT elevation at week 12 (upper limit of normal, ULN; ULN standards were based on the reported articles) with HBsAg seroclearance was reported in 13, 3, 8, 4, and 2 studies, respectively.

Table 2 provides a summary of the pooled estimates for on-treatment factors associated with HBsAg seroclearance, reported in two or more studies. The results showed that lower HBsAg levels (log_10_ IU/mL) at week 12 and 24 were associated with a higher likelihood of achieving HBsAg seroclearance (OR = 0.384, 95% CI 0.230–0.642, *I*^*2*^ = 63.7%; OR = 0.294, 95% CI 0.217–0.398, *I*^*2*^ = 14.2%, respectively). Moreover, a greater reduction in HBsAg (log_10_ IU/mL) from baseline to week 12 and 24 was associated with increased HBsAg seroclearance (OR = 6.689, 95% CI 4.198–10.660, *I*^*2*^ = 0%; OR = 6.513, 95% CI 4.626–9.169, *I*^*2*^ = 42.1%). Similarly, HBsAg decline > 1 or > 0.5 log_10_ IU/ml from baseline to week 12 showed a significant association with HBsAg seroclearance (OR = 18.277, 95% CI 2.096–159.396, *I*^*2*^ = 56.9%; OR = 4.530, 95% CI 3.692–5.558, *I*^*2*^ = 0%). ALT elevation at week 12 was also a strong predictor (OR = 3.622, 95% CI 2.015–6.512, *I*^*2*^ = 0%) (Supplementary Figs. 4, 5).

**Table 2:** Response guided therapy (RGT) predictors associated with HBsAg seroclearance

Predictors	HBsAg seroclearance		
	No. study	OR (95% CI)	*p* value
HBsAg at week 12 (log_10_ IU/mL)	5	0.384 (0.230, 0.642)	< 0.001*
PegIFNα treatment ≤ 48 weeks	3	0.334 (0.168, 0.664)	0.002*
PegIFNα treatment > 48 weeks	2	0.545 (0.156, 1.911)	0.343
HBsAg at week 24 (log_10_ IU/mL)	6	0.294 (0.217, 0.398)	< 0.001*
PegIFNα treatment ≤ 48 weeks	3	0.270 (0.179, 0.408)	< 0.001*
PegIFNα treatment > 48 weeks	3	0.357 (0.185, 0.687)	0.002*
HBsAg at week 48 (log_10_ IU/mL)	2	0.113 (0.009, 1.371)	0.087
HBsAg at week 12 (IU/mL)	3	0.993 (0.983, 1.004)	0.234
HBsAg decline (log_10_ IU/mL)			
From baseline to week 12	3	6.689 (4.198, 10.660)	< 0.001*
PegIFNα treatment ≤ 48 weeks	2	7.196 (4.209, 12.305)	< 0.001*
PegIFNα treatment > 48 weeks	1	–	–
From baseline to week 24	5	6.513 (4.626, 9.169)	< 0.001*
PegIFNα treatment ≤ 48 weeks	3	9.293 (5.699, 15.154)	< 0.001*
PegIFNα treatment > 48 weeks	2	4.632 (2.870, 7.477)	< 0.001*
From baseline to week 12			
≥ 1 versus < 1 log_10_ IU/Ml	2	18.277 (2.096, 159.396)	0.009*
≥ 0.5 versus < 0.5 log_10_ IU/mL	2	4.530 (3.692, 5.558)	< 0.001*
ALT elevation at week 12 (ULN)	2	3.622 (2.015, 6.512)	0.001*

* Significantly associated with HBsAg seroclearance

### Subgroup analysis based on PegIFNα treatment course and region

Subgroup analyses were conducted based on whether the PegIFNα treatment course exceeded the standard 48-week treatment. For baseline characteristics, there was no significant association between age and HBsAg seroclearance for treatment ≤ 48 weeks (OR = 0.978, 95% CI 0.933–1.026, *I*^*2*^ = 65.4%). However, for treatment > 48 weeks, a younger age was associated with a higher likelihood of achieving HBsAg seroclearance (OR = 0.917, 95% CI 0.875–0.961, *I*^*2*^ = 0%). The remaining results were consistent with the overall analysis.

For on-treatment factors, HBsAg at week 12 (log_10_ IU/mL) was significantly associated with HBsAg seroclearance for treatment ≤ 48 weeks (OR = 0.334, 95% CI 0.168–0.664, *I*^*2*^ = 70%), but this association disappeared when the treatment exceeded 48 weeks (OR = 0.545, 95% CI 0.156–1.911, *I*^*2*^ = 74.6%). The remaining results were consistent with the overall analysis (Table 2).

The study included a predominantly Asian population, and similar results were observed in subgroup analysis based on the Asian population as compared to the overall population (Tables 3, 4).

**Table 3:** Baseline guided therapy (BGT) predictors associated with HBsAg seroclearance in Asian population

Predictors	HBsAg seroclearance		
	No. study	OR (95% CI)	*p* value
Age (year)	7	0.951 (0.915, 0.988)	0.01*
PegIFNα treatment ≤ 48 weeks	5	0.967 (0.918, 1.019)	0.208
PegIFNα treatment > 48 weeks	2	0.917 (0.875, 0.961)	< 0.001*
Gender (male vs. female)	2	0.537 (0.371, 0.777)	0.001*
Baseline HBsAg (log_10_ IU/mL)	12	0.421 (0.309, 0.574)	< 0.001*
PegIFNα treatment ≤ 48 weeks	8	0.401 (0.270, 0.595)	< 0.001*
PegIFNα treatment > 48 weeks	4	0.462 (0.251, 0.850)	0.013*
Baseline HBeAg (log S/CO)	2	0.877 (0.556, 1.384)	0.574
Baseline HBV DNA (log_10_ IU/mL)	3	0.416 (0.246, 0.702)	0.001*
PegIFNα treatment ≤ 48 weeks	1	–	–
PegIFNα treatment > 48 weeks	2	0.531 (0.297, 0.950)	0.033*

* Significantly associated with HBsAg seroclearance

**Table 4:** Response guided therapy (RGT) predictors associated with HBsAg seroclearance in Asian population

Predictors	HBsAg seroclearance		
	No. study	OR (95% CI)	*p* value
HBsAg at week 12 (log_10_ IU/mL)	5	0.384(0.230,0.642)	< 0.001*
PegIFNα treatment ≤ 48 weeks	3	0.334(0.168,0.664)	0.002*
PegIFNα treatment > 48 weeks	2	0.545(0.156,1.911)	0.343
HBsAg at week 24 (log_10_ IU/mL)	6	0.294(0.217,0.398)	< 0.001*
PegIFNα treatment ≤ 48 weeks	3	0.270(0.179,0.408)	< 0.001*
PegIFNα treatment > 48 weeks	3	0.357(0.185,0.687)	0.002*
HBsAg at week 48 (log_10_ IU/mL)	2	0.113(0.009,1.371)	0.087
HBsAg at week 12 (IU/mL)	3	0.993(0.983,1.004)	0.234
HBsAg decline (log_10_ IU/mL)			
From baseline to week 12	3	6.689(4.198,10.660)	< 0.001*
PegIFNα treatment ≤ 48 weeks	2	7.196(4.209,12.305)	< 0.001*
PegIFNα treatment > 48 weeks	1	–	–
From baseline to week 24	5	6.513(4.626,9.169)	< 0.001*
PegIFNα treatment ≤ 48 weeks	3	9.810(5.119,18.798)	< 0.001*
PegIFNα treatment > 48 weeks	2	4.632(2.870,7.477)	< 0.001*
HBsAg decline from baseline to week 12			
≥ 1 versus < 1log_10_ IU/mL	1	–	–
≥ 0.5 versus < 0.5 log_10_ IU/mL	2	4.530(3.692,5.558)	< 0.001*
ALT elevation at week 12 (ULN)	2	3.622(2.015,6.512)	0.001*

* Significantly associated with HBsAg seroclearance

Some predictive factors related to HBsAg seroclearance could not be combined due to only one study reporting usable data. These predictors included virological indicators, such as quantitative hepatitis B core antibody (qAnti-HBc), HBsAb, hepatitis B core-related antigen (HBcrAg), HBsAg composition (large hepatitis B surface proteins [LHBs], middle hepatitis B surface proteins [MHBs]); biochemical indicators, such as creatinine (Cr), hemoglobin (HGB), and platelet (PLT); immunological and other biomarkers, such as monocyte Myeloid-derived suppressor cells (mMDSCs), CD4^+^ Treg, CD86^+^ plasmacytoid dendritic cell (pDC), C–X–C Motif Chemokine Ligand 9 (CXCL9), microRNAs-3960, microRNAs-126-3p, and others (Supplementary Table 5).

### Meta-regression, sensitivity and publication bias analysis

Meta-regression was used to explore the effects of 5 confounding factors (publication year, region, study design, treatment naïve and treatment duration) on heterogeneity. Only region was significantly associated with the high heterogeneity of findings on baseline HBsAg (Supplementary Fig. 6). The Cochrane's *Q* test *p* values and *I*^*2*^ statistics indicated high heterogeneity for the factors of HBsAg at week 48 (log_10_ IU/mL) and HBsAg at week 12 (IU/mL) (*I*^*2*^ = 90%, *p* = 0.002; *I*^*2*^ = 81.8%, *p* = 0.004, respectively). However, due to the limited number of studies on these two factors, sensitivity or subgroup analyses could not be conducted. The *I*^*2*^ value for HBsAg decline from baseline to week 12 (log_10_ IU/mL) decreased from 95 to 0% after excluding one study. This change might be attributed to the inclusion of both total HBsAg and HBsAg components in the multifactorial regression analysis. To assess publication bias, Begg or Egger tests were performed for age and baseline HBsAg (log_10_ IU/mL) in studies with seven or more inclusions. The tests did not indicate significant publication bias (Supplementary Figs. 1, 3). However, the Egger test for HBsAg level (log_10_ IU/mL) showed evidence of publication bias (*p* < 0.05), although no literature was identified for correction using the pruning method. Nevertheless, sensitivity analyses did not show any significant changes in the results.

## Discussion

Complete eradication or sterilizing cure of CHB, defined as the elimination of cccDNA and integrated HBV DNA, is considered an unrealistic endpoint under existing medical conditions. However, functional cure is considered an ideal, achievable endpoint and the goal to strive for. As evidenced by multiple studies, aggressive antiviral therapy aimed at achieving functional cure significantly reduces the incidence of cirrhosis, liver decompensation, and liver cancer-related mortality [9, 37]. Expanding the treatment indications for CHB and increasing the coverage of available treatments are highly effective and cost-effective strategies to reduce adverse outcomes related to CHB [38]. Combining NAs with PegIFNα and extending the treatment duration in current therapeutic regimens have been shown to prolong quality-adjusted life-years, making it more cost-effective than NAs monotherapy [39]. Ongoing real-world studies with large sample sizes and multicenter participation are focused on achieving functional cure for CHB patients and reducing the incidence of liver cancer [15, 40, 41].

Nevertheless, functional cure of CHB remains challenging, and most new drugs are still in early clinical development stages. Notably, GSK3228836, an antisense oligonucleotide, has reached Phase III clinical trials, with Phase II results showing a 30% HBsAg seroclearance rate at 24 weeks, but this rate decreased to about 10% after 24 weeks of follow-up, indicating limited durability [42, 43]. Besides lack of durability, the dose of new drugs required also is unclear. To date, many new drugs have been discontinued in clinical stages I/II due to unsatisfying efficacy or safety issues [44]. Therefore, PegIFNα-based therapy remains the preferred choice for achieving functional cure and sustained response after discontinuation.

The investigation of predictors associated with HBsAg seroclearance achieved through PegIFNα treatment for chronic HBV infection holds great clinical significance and health economic implication. Past two decades have witnessed progressive improvement in achieving functional cure with better stratification by certain predictors or biomarkers. In 2004 and 2005, two large-scale, multicenter, randomized trials reported low rates of HBsAg seroclearance or seroconversion (around 3%) induced by 48-week PegIFNα-based therapy in treatment-naive patients [45, 46]. Subgroup analyses from OSST trial and NEW SWITCH study revealed that NA-experienced patients with HBeAg loss and HBsAg < 1500 IU/ml at baseline achieved a markedly higher rate of HBsAg seroclearance (22.2–26.5%) at week 48 after switching to PegIFNα treatment than those who had a baseline HBsAg level of ≥ 1500 IU/ml (1.6–3.8%) [27, 28]. In addition to baseline characteristics (age, gender, genotype, HBsAg level, etc.), on-treatment dynamics of certain factors also showed great potential to be a promising predictor for HBsAg seroclearance during PegIFNα-based therapy. A significantly higher rate of HBsAg seroclearance was observed in patients with week 12 HBsAg < 200 IU/ml (51.4–77.8%) [27, 28], or HBsAg decline from baseline to week 12 > 0.5 log_10_ IU/ml (80.0%), or ALT ≥ 2 ULN during the first 12 weeks (54.8%) [29]. It is worth noting that several studies are exploring the combination of new drugs with PegIFNα treatment [47, 48]. With individualized stratification by proven predictors or biomarkers, clinical trials of new drugs combined with PegIFNα, especially phase 2/3 studies, would result in an evidently higher rate of HBsAg seroclearance and better durability of off-treatment responses than monotherapy.

Based on these findings mentioned above, it is a practical way to significantly improve PegIFNα-induced functional cure rate by selecting “advantaged population” who are the most likely to achieve HBsAg seroclearance according to some characteristics or factors of CHB patients. However, there are still some problems to be solved. Given that one single factor usually has a limited ability to predict treatment responses to PegIFNα, an integrated predictive model consisting of several factors are expected to provide more accurate prediction in PegIFNα treatment. Our study has made important contributions for developing this kind of predictive model and increasing functional cure rates. Firstly, information on predictors for HBsAg seroclearance is dispersed across a number of different individual studies. To our knowledge, this study is the first systematic review and meta-analysis that summarizes information about these predictors through a comprehensive and precise literature search. In our meta-analysis, researchers can obtain comprehensive information on these predictors at the fastest speed, helping with establishment of a more accurate integrated predictive model. Secondly, different studies sometimes drew controversial conclusions on one identical factor. This kind of inconsistent conclusions make researchers confused and pose a great challenge in the process of establishing an integrated predictive model. In this context, we provided our answer to these controversial factors by the means of meta-analysis. In addition, we believe that our answer is more reliable and scientific than any single study on predictors, because meta-analysis can increase the sample size by merging homogeneous studies, enhance the statistical test efficacy, and help to come to a consensus on controversial factors. Lastly, the present study reported a number of reliable predictors for HBsAg seroclearance by PegIFNα, which paved the way for establishment of a more accurate integrated predictive model to identify “advantaged population” and significantly improve functional cure rates. Therefore, our work is conducive to guiding individualized treatment and achieving the highest cost-effectiveness of PegIFNα.

Among the studies included in this meta-analysis, 62.9% were prospective trials, and 48.1% were multi-centre studies. The evaluation of literature quality demonstrated the reliability of the included studies. The findings of this study have revealed several baseline characteristics, such as HBV DNA, HBsAg level, age, and the combination of PegIFNα with NAs therapy, along with on-treatment indicators such as HBsAg levels at weeks 12 and 24, and ALT elevation at week 12, as independent predictors of HBsAg seroclearance, which aligns well with clinical practices. Interestingly, Liu et al. [21] reported that compared to interferon monotherapy, NAs add-on interferon had a similar outcome in their meta‑analysis, while we found that the combination of PegIFNα with NAs resulted in a higher probability of achieving HBsAg seroclearance compared to PegIFNα monotherapy. This disagreement could be attributed to different research objectives and inclusion criteria. We aimed to summarize potential predictors for HBsAg seroclearance by PegIFNα in CHB patients, so only studies that utilized multivariate Cox or logistic regression to report at least one predictor for HBsAg seroclearance by PegIFNα-containing therapy would be included in our meta‑analysis. By contrast, a number of studies, in which conventional interferon-α was used for treating CHB patients, and multivariate Cox or logistic regression was not applied in statistical analysis, were also included in the meta-analysis of Liu et al. Subgroup analysis revealed that there was no significant association between HBsAg at week 12 and HBsAg seroclearance when the treatment duration exceeded 48 weeks; however, HBsAg levels at week 24 may serve as a better predictor. Consequently, the recommended time point for prediction should be shifted to week 24 in PegIFNα treatments lasting longer than 48 weeks. Li et al. demonstrated that in CHB patients whose HBsAg levels plateaued after the initial PegIFNα treatment, adopting a PegIFNα-based intermittent approach could result in a high HBsAg seroclearance rate of up to 44.06% when an early HBsAg response was achieved in both initial treatment and retreatment. Notably, HBsAg levels before retreatment and a decline of > 0.5 log_10_ IU/mL in HBsAg levels at week 12 were identified as independent predictors of HBsAg seroclearance after retreatment [23]. Several studies have shown that prolonging PegIFNα-based therapy based on the treatment response may enhance the likelihood of achieving a functional cure in more patients [22, 49]. To maintain HBsAg seroclearance effectively, it is advisable to ensure a higher HBsAb level at the time of discontinuation [50–52]. In addition, PegIFNα consolidation treatment for 12–24 weeks after HBsAg seroclearance has been found to enhance the durability of functional cure [53]. The guideline in China recommends extending PegIFNα treatment courses based on the patient's condition. However, it is important to note that the majority of studies included in this meta-analysis investigated PegIFNα treatment durations of ≤ 48 weeks. Therefore, the generalizability of the results to patients undergoing longer treatment courses remains limited, and further studies are warranted to facilitate a comprehensive summary analysis.

An increasing number of studies have focused on the multidimensional prediction of HBsAg seroclearance, incorporating immunological, biochemical, and virological indicators. Notably, higher sensitivity quantitative indicators, such as qHBsAg and qHBcrAg, have shown promise in accurately predicting HBsAg seroclearance. Immunological-related indicators, including mMDSCs, CD4^+^ Treg, CD86^+^ pDC, and CXCL9, offer a more comprehensive understanding of antiviral treatment mechanisms, thereby potentially leading to further improvements in functional cure rates.

There are some limitations in the present study. To begin with, this meta-analysis did not conduct subgroup analyses on potential factors that may influence HBsAg seroclearance, such as disease stage, prior antiviral treatment (treatment-naive or NAs-experienced), and various treatment strategies (initial combination, sequential combination, extended or intermittent treatment). The limited number of studies and patients on these factors was a constraint. Moreover, studies on predictors of HBsAg seroclearance in special groups, such as children and postpartum women, were also limited. To reduce heterogeneity caused by these populations, the inclusion criteria for this meta-analysis were restricted to adults diagnosed with chronic HBV infection, and the majority of the studies included were from Asian populations, with most of them not measuring the genotype. Only one multicenter study compared the relationship of genotype B or C with HBsAg seroclearance [54]. Last but not the least, due to the nature of methodology, our study revealed associations of these identified factors with HBsAg seroclearance by PegIFNα-based regimens but did not establish causalities. We will try to investigate causalities between them in future RCTs.

In conclusion, this is the first meta-analysis to identify predictors of HBsAg seroclearance with PegIFNα-based regimens, including baseline factors (age, gender, genotype, combination therapy, HBV DNA, and HBsAg levels), and on-treatment factors (HBsAg levels, HBsAg reduction and ALT elevation), which is valuable in developing a better integrated predictive model for HBsAg seroclearance to guide individualized treatment and achieve the highest cost-effectiveness of PegIFNα.

### Supplementary Information

Below is the link to the electronic supplementary material.

Supplementary file1 (DOCX 115443 KB)

## Data Availability

The data presented in this study are available in article or supplementary material.
