# Supplementary material for: Predictors of HBsAg seroclearance in patients with chronic HBV infection treated with pegylated interferon-α: a systematic review and meta-analysis
Source: Hepatol Int. 2024 Mar 9;18(3):892–903. doi: 10.1007/s12072-024-10648-8 (PMC11126512; doi:10.1007/s12072-024-10648-8)
Supplement: Supplementary file 1 — Supplementary file1 (DOCX 115443 KB) [file 12072_2024_10648_MOESM1_ESM.docx]

**Supplementary Table 1. Searching strategy and result.**

**Number of citations by each database searched**

| **Databases** | **Citations** |
| --- | --- |
| PubMed | 722 |
| Embase | 1633 |
| Cochrane Library | 857 |
| Web of science | 1138 |
| Total databases | 4350 (with 1381 duplicates) |
| Total databases after removing duplicates | 2969 |

Searching strategy for Pubmed

1."hepatitis b, chronic" [MeSH Terms]

2. "Hepatitis B Surface Antigens" [Mesh]

3. "Seroconversion" [Mesh]

4. (((((Hepatitis B, Chronic [Title/Abstract]) OR (Chronic Hepatitis B Virus Infection [Title/Abstract])) OR (Chronic Hepatitis B [Title/Abstract])) OR (Hepatitis B Virus Infection, Chronic [Title/Abstract])) OR (HBV [Title/Abstract])) OR (hepatitis B [Title/Abstract])

5. (((Hepatitis B Surface Antigens [Title/Abstract]) OR (HBsAg [Title/Abstract])) OR (Australia Antigen [Title/Abstract])) OR (Antigen, Australia [Title/Abstract])

6. ((((((((Seroconversion [Title/Abstract]) OR (Seroconversions [Title/Abstract])) OR (Clearance [Title/Abstract])) OR (Undetectable [Title/Abstract])) OR (Seroreversion [Title/Abstract])) OR (seroclearance [Title/Abstract])) OR (loss [Title/Abstract])) OR (disappearance [Title/Abstract])) OR (functional cure [Title/Abstract])

7. #1 OR #4

8.#2 OR #5

9.#3 OR #6

10.#7 AND #8

11.#10 AND #9

12. "Clinical Trial" [Publication Type]

13. "Clinical Trials as Topic" [Mesh]

14. ((((((Clinical Trial [Title/Abstract]) OR (Clinical Trials as Topic [Title/Abstract])) OR (Intervention Study [Title/Abstract])) OR (clinic trial [Title/Abstract])) OR (controlled clinical trial [Title/Abstract])) OR (trial [Title/Abstract])) OR (clinical study [Title/Abstract])

15.#12 OR #13 OR #14

16.#15 AND #11

Searching strategy for Embase

1.'chronic hepatitis b'/exp

2. "Hepatitis B Surface Antigens" [Mesh]

3. 'seroconversion'/exp

4. 'hepatitis b, chronic:ab,ti OR 'chronic hepatitis b virus infection':ab,ti OR 'chronic hepatitis b':ab,ti OR 'hepatitis b virus infection, chronic':ab,ti OR hbv:ab,ti OR 'hepatitis b':ab,ti

5. 'hepatitis b surface antigens:ab,ti OR hbsag:ab,ti OR 'australia antigen':ab,ti OR 'antigen, australia':ab,ti

6. seroconversion:ab,ti OR seroconversions:ab,ti OR clearance:ab,ti OR undetectable:ab,ti OR seroreversion:ab,ti OR seroclearance:ab,ti OR loss:ab,ti OR disappearance:ab,ti OR 'functional cure':ab,ti

7. #1 OR #4

8. #2 OR #5

9. #3 OR #6

10. #7 AND #8 AND #9

11. 'clinical trial'/exp

12. 'clinical trial':it OR 'clinical trials as topic: ab,ti OR 'clinical trial':ab,ti OR 'intervention study':ab,ti OR 'clinic trial':ab,ti OR 'controlled clinical trial':ab,ti OR trial:ab,ti OR 'clinical study':ab,ti

13. #11 OR #12

14. #10 AND #13

### Searching strategy for Web of science

1.Hepatitis B, Chronic (Topic) OR Chronic Hepatitis B Virus Infection (Topic) OR Chronic Hepatitis B (Topic) OR Hepatitis B Virus Infection, Chronic (Topic) OR HBV(Topic) OR hepatitis B (Topic)

2. Hepatitis B Surface Antigens (Topic) OR HBsAg (Topic) OR Australia Antigen (Topic) OR Antigen, Australia (Topic)

3. Intervention Study (Topic) OR clinic trial (Topic) OR controlled clinical trial (Topic) OR trial (Topic) OR clinical study (Topic) OR Clinical Trials as Topic (Topic) OR Clinical Trial (Topic) OR Clinical Trial (Publication Titles)

4. functional cure (Topic) OR Seroconversions (Topic) OR Clearance (Topic) OR Undetectable (Topic) OR Seroreversion (Topic) OR seroclearance (Topic) OR loss (Topic) OR disappearance (Topic)

5. #4 AND #3 AND #2 AND #1

Searching strategy for Cochrane Library

1.MeSH descriptor: [Hepatitis B, Chronic] explode all trees

2. (Hepatitis B, Chronic): ti,ab,kw OR (Chronic Hepatitis B Virus Infection):ti,ab,kw OR (Chronic Hepatitis B):ti,ab,kw OR (Hepatitis B Virus Infection, Chronic):ti,ab,kw OR (HBV):ti,ab,kw

3. (hepatitis B): ti,ab,kw

4.#1 or #2 or #3

5.MeSH descriptor: [Hepatitis B Surface Antigens] explode all trees

6. (Hepatitis B Surface Antigens): ti,ab,kw OR (HBsAg):ti,ab,kw OR (Australia Antigen):ti,ab,kw OR (Antigen, Australia):ti,ab,kw

7.MeSH descriptor: [Seroconversion] explode all trees

8. (Seroconversion): ti,ab,kw OR (Seroconversions):ti,ab,kw OR (Clearance):ti,ab,kw OR (Undetectable):ti,ab,kw OR (Seroreversion):ti,ab,kw

9. (seroclearance): ti,ab,kw OR (loss):ti,ab,kw OR (disappearance):ti,ab,kw OR (functional cure):ti,ab,kw

10.#5 or #6

11.#7 or #8 or #9

12.MeSH descriptor: [Clinical Trial] explode all trees

13.MeSH descriptor: [Clinical Trials as Topic] explode all trees

14. (Clinical Trials):pt OR (Clinical Trials as Topic): ti,ab,kw OR (Clinical Trials):ti,ab,kw OR (Intervention Study):ti,ab,kw OR (clinic trial controlled):ti,ab,kw

15. (clinical trial): ti,ab,kw OR (trial):ti,ab,kw OR (clinical study):ti,ab,kw

16.#12 or #13 or #14 or #15

17.#4 and #10 and #11 and #16

**Supplementary Table 2. Characteristics of the studies including in meta-analysis**

| **Study/year** | **Design** | **Country** | **Treatment and Duration (Weeks)** | **Follow-up (weeks)** | **No.**  **Total/HS** | **Age (year)** | **Male/**  **Female** | **Before PegIFNα** | | | |
| --- | --- | --- | --- | --- | --- | --- | --- | --- | --- | --- | --- |
|  |  |  |  |  |  |  |  | **HBeAg (+/-)** | **HBsAg**  **(log_10_ IU/mL)** | **ALT (U/L)** | **HBV DNA**  **(log_10_ IU/mL)** |
| Marcellin P 2016(1) | RCT | France | (PegIFNα+NAs) *48 (PegIFNα+NAs) *16+NAs *32 NAs *120 PegIFNα *48 | 72 | 186/19 184/1 185/0 185/5 | 38±16.7 37±9 36±10.9 38±10.5 | 127/59 119/65 121/64 119/66 | 108/78 105/79 109/76 106/79 | 3.9±0.8 3.8±0.9 3.9±0.8 3.8±0.8 | 121±181 112±94 101±68 107±92 | 7.1±1.5 7.1±1.5 7.0±1.5 6.9±1.6 |
| Bao X 2020(2) | SCS | China | (PegIFNα+NAs) *102.6±56.1 | 96 | 42/42 | 37.3±11.4 | 30/12 | 24/18 | + | 122.7 (27.0-336.0) | 5.2±2.2 |
| Buster EH 2009(3) | RCT | Netherlands | ((PegIFNα+NAs)/PegIFNα)) *52 | 144 | 172/19 | 35.5±13.3 | 137/35 | + | + | 4.7±4.0 | log_10_ copies/ml:9.0±0.9 |
| Cao W 2022(4) | SCS | China | PegIFNα±NAs * (24~48) | 192 | 63/17 | 30 (20–51) | 38/25 | + | 3.857±0.692 | 253.300 (129.600, 361.100) | 7.059 ±1.161 |
| Cao Z 2017(5) | DCS | China | (PegIFNα±NAs) *96 Untreated | 0 | 94/30 40/0 | 38.8±10.0 39.8±10.6 | 62/32 26/14 | - | <3 | 28.3±9.0 25.4±8.4 | <3.301 |
| Chu JH 2022(6) | SCS | China | (PegIFNα+NAs) *48 | 0 | 2579/947 | 41.2±8.8 | 2086/493 | - | 2.3±1.2 | 26±22.0 | <2 or undetected |
| Erken R 2022(7) | SCS | Netherlands | ((PegIFNα+NAs)/Untreated)) *48 | 240 | (105/19)/ (13/4) | 43.1±10.9 | 75/49 | 14/110 | 3.4±0.9 | 29 (22–54) | 3.8±2.2 |
| Huang D 2022(8) | RCT | China | (PegIFNα+NAs) *48+PegIFNα *48 | 24 | 80/24 | 34 (26-46) | 66/12 | 33/47 | 2.797± 0.047 | 20.0 (14.0-29.0) | 1.390 ±0.038 |
| Lee IC 2021(9) | SCS | China | PegIFNα *48 | 260 | 57/6 | 48.0±10.4 | 47/10 | - | 3.07± 0.8 | 188±174 | 5.91±1.39 |
| Lee IC 2018(10) | SCS | China | PegIFNα *48 | 360 | 233/33 | 44.6±10.1 | 206/27 | - | + | 145±129 | 5.59±1.61 |
| Li GJ 2015(11) | DCS | China | (PegIFNα+NAs) *48 NAs *48 | 0 | 81/9 116/0 | 31.5 (23–54) 33 (23–53) | 31/19 31/19 | S/CO:  2.0 (0.05–3.2) 2.1 (1.0–3.3) | 3.6 (2.8–4.5) 3.8 (2.9–4.0) | 31.5 (11–149) 24.5 (10–174) | <3 <3 |
| Li M 2022(12) | SCS | China | (PegIFNα+NAs) * (60~72) | 48 | 304/59 | 37.97±83 | 215/89 | 231/73 | 3.37±0.85 | NR | NR |
| Lin X 2022(13) | SCS | China | PegIFNα *96 | 0 | 60/60 | 38.95±11.23 | 43/17 | - | IU/mL:  198.78±236.54 | 31.31±14.51 | <3.301 |
| Takkenberg RB 2013(14) | SCS | Netherlands | (PegIFNα+NAs) *48 | 96 | 92/13 | 39.61±10.23 | 68/24 | 44/48 | 3.46± 0.6 | ULN:  1.9 (1.1–2.9) | 5.6 (1.1) |
| Thanapirom K 2019(15) | SCS | Thailand | PegIFNα *48 | 24 | 107/6 | 47.0±9.5 | 82/25 | - | 3.28±0.56 | 92.8±84.5 | 5.61±1.53 |
| Wang WX 2022(16) | DCS | China | (PegIFNα+NAs) *48 NAs *48 | 0 | 59/17 15/0 | 39 (32-47) 38.0 (34.0, 47.5) | 49/10 13/2 | 18/41 6/9 | 2.59 (1.97, 2.95) 2.73 (2.21, 3.01) | 22.0 (18.0, 27.0)  20.0 (14.0, 30.5) | - |
| Wu FP 2021(17) | DCS | China | PegIFNα *48 Untreated | 24 | 142/68 156/3 | 37.9±10.7 36.6±10.6 | 96/46 104/52 | - | ≤3.176 | 25.5 (16.0, 35.0) 22.0 (16.0, 32.0) | <3.301 or undetected |
| Wu FP 2020(18) | DCS | China | (PegIFNα+NAs) *48 NAs *48 | 24 | 91/34 104/2 | 38.11±9.74 37.34±11.01 | 62/29 76/28 | - | ≤ 3.176 | 24 (8.0-73) 27 (7-66) | <2 |
| Yan L 2018(19) | DCS | China | (PegIFNα+NAs) *48 PegIFNα *48 NAs *48 | 24 | 26/4 22/2 53/0 | 43.17 (27–60) 35.2 (25–50) 45.6 (28–65) | 24/2 21/1 37/16 | - | IU/ml:1219±1736 1250±1738 1898 ±1317 | 31.83±14.16 32.45±13.73 29.8 ±12.98 | - |
| Yang X 2022(20) | SCS | China | (PegIFNα+NAs) *48 | 0 | 292/65 | 44 (38-49) | 263/29 | - | IU/mL:268.1 (80.77-682.16) | 25 (20-34) | - |
| Yang Y 2018(21) | DCS | China | (PegIFNα±NAs) * (48~96) | 0 | 67/27 | 38.47±9.62 | 55/12 | - | < 3.176 | ULN: ≤2 | < 3 |
| Zhang W 2022(22) | DCS | China | (PegIFNα±NAs) * (24~48)(IHC)  (PegIFNα±NAs) * (24~48)(CHB) | 24 | 119/56 209/80 | 36 (31-42) 40 (33.-46.5) | 87/32 159/50 | - | IU/mL:29.10 (5.10-229.89) 159.11 (32.90-406.44) | 20.0 (15.0-28.0) 21.0 (15.0-29.0) | <3.301 |
| Zheng C 2019(23) | DCS | China | PegIFNα *48 (PegIFNα +NAs) *48 | 0 | 66/2 77/10 | 29.6±5.5 30.3±7.3 | 48/18 | COI/mL:482.9±535.3 379.7±521.1 | 3.7±0.8 3.6±1.2 | 198.3±222.9 236.4±292.4 | 6.9±1.1 6.6±1.7 |
| Zhang M 2020(24) | RCT | China | PegIFNα *48 | 24 | 727/21 | 28.2 ± 0.3 | 523/204 | + | 4.3 ± 0.02 | IU/mL:193.0±5.5 | 7.9 ± 0.03 |
| Zhang Y 2023(25) | SCS | China | PegIFNα *48 | 0 | 97/24 | 28.5±2.8 | 74/23 | - | + | 37.27±8.92 | + |
| Jia H 2023(26) | RCT | China | (PegIFNα+NAs + GM-CSF + HBV vaccine) *48 (PegIFNα+NAs) *48 NAs *48 | 24 | 95/39 92/26 100/0 | 37.08±9.30 39.23±9.28 39.68±8.03 | 75/20 71/21 76/24 | - | 2.69±0.77 2.74±0.47 2.71±0.69 | 23.80±8.40 24.00±8.75 23.75±9.43 | - |
| Ren P 2021(27) | SCS | China | (PegIFNα±NAs) *48 | 0 | 320/39 | 37 (31-43) | 269/51 | 107/213 | 3.04 (2.63-3.45) | 23 (19-32) | <3 |

SCS,single cohort study; DCS,double cohort study; Follow-up,follow up after PegIFNα cessation; NAs,nucleos(t)ide analogues; HS,HBsAg seroclearance; “+”, positive; “-”,negative or lower detection limit; NR,Not Report.

**Supplementary Table 3. Quality assessment of cohort Studies**

| **Study** | **Selection** | | | | **Comparability of subjects** | **Outcomes** | | | **Total** |
| --- | --- | --- | --- | --- | --- | --- | --- | --- | --- |
|  | **Representative of exposed cohort** | **Selection of non-exposed cohort** | **Ascertainment of exposure** | **Demonstration outcome not present at start of study** |  | **Outcome assessment** | **Adequate follow-up for outcomes** | **Adequate follow-up of cohorts** |  |
| Cao Z 2017 | 1 | 1 | 1 | 1 | 2 | 1 | 1 | 1 | 9 |
| Li GJ 2015 | 1 | 1 | 1 | 1 | 2 | 1 | 1 | 1 | 9 |
| Wang WX 2022 | 1 | 1 | 1 | 1 | 2 | 1 | 1 | 0 | 8 |
| Wu FP 2021 | 1 | 1 | 1 | 1 | 2 | 1 | 1 | 1 | 9 |
| Wu FP 2020 | 1 | 1 | 1 | 1 | 2 | 1 | 1 | 1 | 9 |
| Yan L 2018 | 1 | 1 | 1 | 1 | 1 | 1 | 1 | 0 | 7 |
| Zhang W 2022 | 1 | 1 | 1 | 1 | 1 | 1 | 1 | 0 | 7 |
| Zheng C 2019 | 1 | 1 | 1 | 1 | 2 | 1 | 1 | 0 | 8 |
| Yang Y 2018 | 1 | 1 | 1 | 1 | 2 | 1 | 1 | 0 | 8 |

**Supplementary Table 4. Quality assessment of single arm studies**

| **Study** | **A clearly stated aim** | **Inclusion of consecutive patients** | **Prospective collection of data** | **Endpoints appropriate to the aim of the study** | **Unbiased assessment of the study endpoint** | **Follow-up period appropriate to the aim of the study** | **Loss to follow up less than 5%** | **Prospective calculation of the study size** | **Total** |
| --- | --- | --- | --- | --- | --- | --- | --- | --- | --- |
| Bao X 2020 | 2 | 1 | 1 | 2 | 2 | 2 | 1 | 1 | 12 |
| Cao W 2022 | 2 | 2 | 2 | 2 | 2 | 2 | 1 | 1 | 14 |
| Chu JH 2022 | 2 | 2 | 2 | 2 | 2 | 2 | 1 | 1 | 14 |
| Erken R 2022 | 2 | 1 | 1 | 2 | 2 | 2 | 1 | 1 | 12 |
| Lee IC 2021 | 2 | 2 | 2 | 2 | 2 | 2 | 1 | 1 | 14 |
| Lee IC 2018 | 2 | 1 | 1 | 2 | 2 | 2 | 1 | 1 | 12 |
| Li M 2022 | 2 | 2 | 2 | 2 | 2 | 2 | 1 | 1 | 14 |
| Lin X 2022 | 2 | 1 | 1 | 2 | 2 | 2 | 1 | 1 | 12 |
| Takkenberg RB 2013 | 2 | 1 | 1 | 2 | 2 | 2 | 1 | 1 | 12 |
| Yang X 2022 | 2 | 2 | 2 | 2 | 2 | 2 | 2 | 1 | 15 |
| Thanapirom K 2019 | 2 | 1 | 1 | 2 | 2 | 2 | 1 | 1 | 12 |
| Zhang Y 2023 | 2 | 1 | 2 | 2 | 2 | 2 | 2 | 2 | 13 |

**Supplementary Table 5. Summary of the predictors for HBsAg seroclearance of PegIFNα Therapy**

| **Studies** | **Predictors** | **OR/HR (95% CI)** | **P-value** |
| --- | --- | --- | --- |
| Wang WX 2022 | Baseline qAnti-HBc (< 0.1 IU/mL) | 24.83 (2.369, 260.22) | 0.007* |
|  | Baseline HBsAg levels (<400 IU/mL) | 11.516 (1.662, 79.779) | 0.013* |
| Chu JH 2022 | Baseline HBsAb positive | 1.384 (0.862, 2.222) | 0.178 |
|  | Baseline Cr (μmol/L) | 0.999 (0.990, 1.008) | 0.834 |
|  | Baseline HGB (g/L) | 1.001 (0.992, 1.010) | 0.85 |
|  | Baseline Body- mass index | 0.993 (0.951, 1.037) | 0.744 |
|  | Area (East, North, Northeast/Central South) | 0.710 (0.546, 0.923) | 0.011* |
|  | Northwestt/Central South | 0.387 (0.220, 0.682) | 0.001* |
|  | Southwest/Central South | 0.784 (0.557, 1.104) | 0.164 |
|  | ALT decline from baseline to week 12 (>27 U/L) | 1.365 (1.087, 1.715) | 0.008* |
|  | ALB decline from baseline to week 12 (>2.1 g/L) | 1.244 (0.986, 1.570) | 0.066 |
|  | HGB decline from baseline to week 12 (>14 g/L) | 1.558 (1.204, 2.016) | 0.001* |
|  | PLT count decline from baseline to week 12 (>86*10^9^/L) | 1.144 (0.889, 1.472) | 0.294 |
| Lee IC 2021 | Baseline CXCL9 (>200 vs <200 pg/mL) | 8.154 (1.120, 59.358) | 0.038* |
|  | HBsAg decline from baseline to EOT (>1 log_10_ IU/mL vs <1 log_10_ IU/mL) | 23.296 (2.652, 204.645) | 0.005* |
|  | Baseline HBsAg (<750 vs > 750 IU/mL) | 10.507 (1.163, 94.925) | 0.036* |
| Yang Y 2018 | microRNAs-3960 | 3.053 (1.448, 6.436) | 0.003* |
|  | microRNAs-126-3p | 1.901 (0.984, 3.672) | 0.056 |
| Thanapirom K 2019 | GC rs4588 (CA/AA vs. CC) | 2.16 (0.22, 21.27) | 0.51 |
|  | GC rs222020 (TT vs. CC/CT) | 17.72 (1.07, 294.38) | 0.04* |
| Huang D 2022 | HBcrAg at EOT (log_10_ U/mL) | 0.389 (0.157, 0.963) | 0.041* |
|  | HBsAb at EOT (log_10_ IU/L) | 3.862 (1.389, 10.741) | 0.01* |
|  | HBsAb at week 24 (log_10_ IU/L) | 2.211 (0.678, 7.209) | 0.188 |
|  | HBsAb at week 48 (log_10_ IU/L) | 1.841 (0.746, 4.545) | 0.186 |
|  | HBsAb at week 72 (log_10_ IU/L) | 3.055 (1.203, 7.758) | 0.019* |
|  | HBeAg decline from baseline to week 24 (log_10_ S/CO) | 3.321 (0.333, 33.090) | 0.306 |
|  | HBeAg decline from baseline to week 48 (log_10_ S/CO) | 1.026 (0.213, 4.953) | 0.974 |
|  | HBeAg decline from baseline to week 72 (log_10_ S/CO) | 1.890 (0.443, 8.059) | 0.39 |
|  | HBsAg at week 72 (log_10_ IU/mL) | 0.010 (0.000, 0.864) | 0.043* |
|  | HBsAg decline from baseline to week 48 (log_10_ IU/mL) | 2.875 (1.396, 5.920) | 0.004* |
|  | HBsAg decline from baseline to week 72 (log_10_ IU/mL) | 9.922 (2.055, 47.916) | 0.004* |
|  | Baseline HBcrAg (log_10_ U/mL) | 0.785 (0.446, 1.382) | 0.402 |
|  | HBcrAg at week 24 (log_10_ U/mL) | 0.642 (0.334, 1.234) | 0.184 |
|  | HBcrAg at week 48 (log_10_ U/mL) | 0.594 (0.303, 1.163) | 0.129 |
|  | HBcrAg at week 72 (log_10_ U/mL) | 0.564 (0.262, 1.214) | 0.143 |
|  | HBcrAg decline from baseline to week 24 (log_10_ U/mL) | 1.784 (0.452, 7.047) | 0.409 |
|  | HBcrAg decline from baseline to week 48 (log_10_ U/mL) | 1.925 (0.563, 6.577) | 0.296 |
|  | HBcrAg decline from baseline to week 72 (log_10_ U/mL) | 1.739 (0.583, 5.185) | 0.321 |
|  | HBV DNA at week 24 (log_10_ IU/mL) | 1.808 (0.013, 251.108) | 0.814 |
|  | HBV DNA decline from baseline to week 24 (log_10_ U/mL) | 0.650 (0.046, 9.232) | 0.751 |
|  | HBV DNA decline from baseline to week 48 (log_10_ U/mL) | 0.751 (0.035, 16.220) | 0.855 |
|  | HBV DNA decline from baseline to week 72 (log_10_ U/mL) | 5.886 (0.162, 213.992) | 0.334 |
|  | HBeAg at week 24 (log_10_ S/CO) | 0.568 (0.215, 1.501) | 0.254 |
|  | HBeAg at week 48 log_10_ S/CO) | 0.558 (0.167, 1.861) | 0.342 |
|  | HBeAg at week 72 (log_10_ S/CO) | 0.405 (0.105, 1.564) | 0.19 |
| Erken R 2022 | Baseline ORF X change (Log_10_ fold) | 0.24 (0.08, 0.69) | 0.008* |
| Zheng C 2019 | Hepatic fibrosis | 1.69 (1.65, 1.95) | 0.028* |
| Cao W 2022 | CD86 pDC% | 1.183 (1.021, 1.370) | 0.025* |
| Lin X 2022 | Baseline LHBs (IU/mL) | 0.435 (0.221, 0.854) | 0.016 |
|  | Baseline MHBs (IU/mL) | 0.136 (0.037, 0.502) | 0.003* |
|  | LHBs at week 12 (IU/mL) | 0.137 (0.022, 0.853) | 0.033* |
|  | MHBs at week 12 (IU/mL) | 0.049 (0.003, 0.907) | 0.043* |
|  | LHBs decline from baseline to week 12 (log_10_ IU/mL) | 0.968 (0.932, 1.005) | 0.251 |
|  | MHBs decline from baseline to week 12 (log_10_ IU/mL) | 0.980 (0.948, 1.014) | 0.241 |
| Zhang M 2020 | Baseline Body weight | 0.94 (0.9, 0.98) | 0.005* |
|  | Body weight at week 24 | 0.94 (0.9, 0.98) | 0.005* |
|  | Baseline HBeAg (log_10_ COI) | 3.41 (1.24, 9.4) | 0.018* |
|  | ALT at week 12 (IU/mL) | 1.004 (1.0, 1.007) | 0.043* |
|  | HBeAg at week 12 (log_10_ COI) | 2.2 (1.17, 4.2) | 0.015* |
|  | HBeAg at week 24 (log_10_ COI) | 3.41 (1.24, 9.4) | 0.018* |
| Buster EH 2009 | Baseline ALT (ULN) | 1.00 (0.90, 1.13) | 0.91 |
|  | Baseline Age (per 10-year increase) | 1.63 (1.20, 2.24) | 0.002* |
|  | Baseline HBV DNA (log_10_ copies/mL) | 1.29 (0.75, 2.24) | 0.36 |
|  | HBeAg seroclearance at week 4 | 12.43 (1.63, 95.07) | 0.02* |
|  | HBeAg seroconversion at week 4 | 12.43 (1.63, 95.07) | 0.02* |
|  | HBeAg seroclearance at week 24 | 1.51 (0.43, 5.25) | 0.52 |
|  | HBeAg seroconversion at week 24 | 1.69 (0.48, 5.9) | 0.41 |
|  | HBV DNA at week 24 (< 400 copies/mL) | 5.44 (1.99, 14.90) | 0.001 |
|  | HBeAg seroclearance at week 32 | 27.38 (6.28,119.34) | < 0.001* |
|  | HBeAg seroconversion at week 32 | 9.36 (3.62,24.17) | < 0.001* |
|  | HBV DNA at week 32 (< 400 copies/mL) | 10.09 (3.49,29.20) | < 0.001* |
|  | HBeAg seroclearance (≤week 32) | 9.19 (2.09,40.36) | < 0.001* |
| Wu FP 2021 | Baseline HBV DNA (<20 IU/mL) | 5.434 (3.561,8.669) | 0.001* |
| Li GJ 2015 | Age (<40 years old) | 0.873 (0.148,5.142) | 0.881 |
|  | Previous ETV duration (years) | 3.537 (0.681,18.379) | 0.133 |
| Zhang Y 2023 | Baseline mMDSCs (%) | 2.615 (1.116,6.125) | 0.027 |
|  | Baseline CD4+ Tregs (%) | 1.057 (0.790,1.416) | 0.708 |
| Takkenberg RB 2013 | IFN-treatment naïve | 0.05 (0.02, 3.86) | 0.32 |
| Marcellin P 2016 | Genotype (B vs C) | 1.754 (0.4237, 8.333) | / |
| Ren P 2021 | Baseline HBsAg levels (<1000 IU/mL) | 6.83 (1.86-25.09) | 0.004 |

**Supplementary Figure 1.** Plots of the association between age and HBsAg seroclearance in analyses (A) forest plots; (B) forest plots (Asian population); (C) sensitivity analysis; (D) publication bias of Begg's Test (P=0.536); and (E) publication bias of Egger's Test (P=0.746).

**Supplementary Figure 2.** Forest plots of the association between Gender, Genotype, treatment strategy, Baseline HBeAg, Baseline HBV DNA and HBsAg seroclearance (A) gender (male vs. female); (B) genotype (genotype A vs. B/D); (C)treatment strategy (combination vs. monotherapy); (D) baseline HBeAg (Log S/CO); (E) baseline HBV DNA (log_10_ IU/mL); and (F) baseline HBV DNA (log_10_ IU/mL) in Asian population.

**Supplementary Figure 3.** Plots of the association between baseline HBsAg (log_10_ IU/mL) and HBsAg seroclearance in analyses (A) forest plots; (B) forest plots (Asian population); (C) sensitivity analysis (D) publication bias of Begg's Test (P= 0.101); (E) publication bias of Egger's Test (P= 0.005); and (F) trim-and-fill method.

**Supplementary Figure 4.** Forest plots of the association between HBsAg on treatment and HBsAg seroclearance (A) HBsAg at week 12 (log_10_ IU/mL); (B) HBsAg at week 24 (log_10_ IU/mL); (C) HBsAg at week 48 (log_10_ IU/mL); and (D) HBsAg at week 12 (IU/mL).

**Supplementary Figure5.** Forest plots of the association between HBsAg, ALT and HBsAg seroclearance (A) HBsAg decline (log_10_ IU/mL) from baseline to week 12; (B) HBsAg decline (log_10_ IU/mL) from baseline to week 24; (C) HBsAg decline from baseline to week 12 (≥ 1 vs. < 1 log_10_ IU/ml, (≥ 0.5 vs. < 0.5log_10_ IU/ml); (D) HBsAg decline from baseline to week 12 (≥ 1 vs. < 1 log_10_ IU/ml, ≥ 0.5 vs. < 0.5log_10_ IU/ml)in Asian population; and (E) ALT elevation at week 12 (ULN).

Supplementary Figure 6. Meta-regression of Baseline HBsAg (log10 IU/mL) on publication year, region, study design, treatment naïve and treatment duration. (A) publication year; (B) region (Asia, others); (C) study design (RCTs, observational studies); (D) treatment naïve (naïve, treated and mixed); (E) treatment duration (≤ 48 weeks, > 48 weeks).

**References**

1.	Marcellin P, Ahn SH, Ma X, Caruntu FA, Tak WY, Elkashab M, Chuang WL, Lim SG, Tabak F, Mehta R, et al. Combination of Tenofovir Disoproxil Fumarate and Peginterferon alpha-2a Increases Loss of Hepatitis B Surface Antigen in Patients with Chronic Hepatitis B. Gastroenterology 2016;150:134‐144.e110, 2016

2.	Bao XL, Guo J, Xiong F, Qu YC, Gao Y, Gu N, Lu J. Clinical characteristics of chronic hepatitis B cured by peginterferon in combination with nucleotide analogs. International Journal of Infectious Diseases 2020;96:562-566, Jul, 2020

3.	Buster EH, Flink HJ, Simsek H, Heathcote EJ, Sharmila S, Kitis GE, Gerken G, Buti M, de Vries RA, Verhey E, Hansen BE, Janssen HL. Early HBeAg loss during peginterferon alpha-2b therapy predicts HBsAg loss: results of a long-term follow-up study in chronic hepatitis B patients. Am J Gastroenterol 2009;104:2449-2457, Oct, 2009

4.	Cao W, Xie S, Zhang L, Bi X, Lin Y, Yang L, Lu Y, Liu R, Chang M, Wu S, Shen G, Dong J, Xie Y, Li M. Expression of Functional Molecule on Plasmacytoid Dendritic Cells Is Associated With HBsAg Loss in HBeAg-Positive Patients During PEG-IFN α-2a Treatment. Front Immunol 2022;13:891424, 2022

5.	Cao Z, Liu Y, Ma L, Lu J, Jin Y, Ren S, He Z, Shen C, Chen X. A potent hepatitis B surface antigen response in subjects with inactive hepatitis B surface antigen carrier treated with pegylated-interferon alpha. Hepatology 2017;66:1058-1066, Oct, 2017

6.	Chu JH, Huang Y, Xie DY, Deng H, Wei J, Guan YJ, Li GJ, Zeng YL, Yang JH, Chen XY, Shang J, Li JB, Gao N, Gao ZL. Real-world study on HBsAg loss of combination therapy in HBeAg-negative chronic hepatitis B patients. Journal of Viral Hepatitis 2022;29:765-776, 2022

7.	Erken R, Loukachov V, van Dort K, van den Hurk A, Takkenberg RB, de Niet A, Jansen L, Willemse S, Reesink H, Kootstra N. Quantified integrated hepatitis B virus is related to viral activity in patients with chronic hepatitis B. Hepatology 2022;76:196-206, Jul, 2022

8.	Huang D, Wu D, Wang Y, Wang P, Yuan W, Hu D, Han M, Yan W, Ning Q. End-of-treatment hepatitis b core-related antigen and surface antibody predict durable functional cure to peg-ifn-α in nuc-suppressed chb patients. Hepatology 2021;74:527A, 2021

9.	Lee IC, Su CW, Lan KH, Wang YJ, Lee KC, Lin HC, Hou MC, Huang YH. Virological and immunological predictors of long term outcomes of peginterferon alfa-2a therapy for HBeAg-negative chronic hepatitis B. Journal of the Formosan Medical Association 2021;120:1676-1685, Sep, 2021

10.	Lee IC, Yang SS, Lee CJ, Su CW, Wang YJ, Lan KH, Lin HC, Hou MC, Peng CY, Huang YH. Incidence and predictors of HBsAg loss after peginterferon therapy in HBeAg-negative chronic hepatitis B: A multicenter, long-term follow-up study. Journal of Infectious Diseases 2018;218:1075-1084, 2018

11.	Li GJ, Yu YQ, Chen SL, Fan P, Shao LY, Chen JZ, Li CS, Yi B, Chen WC, Xie SY, Mao XN, Zou HH, Zhang WH. Sequential Combination Therapy with Pegylated Interferon Leads to Loss of Hepatitis B Surface Antigen and Hepatitis B e Antigen (HBeAg) Seroconversion in HBeAg-Positive Chronic Hepatitis B Patients Receiving Long-Term Entecavir Treatment. Antimicrobial Agents and Chemotherapy 2015;59:4121-4128, Jul, 2015

12.	Li M, Xie S, Bi X, Sun F, Zeng Z, Deng W, Jiang T, Lin Y, Yang L, Lu Y, Zhang L, Yi W, Xie Y. An optimized mode of interferon intermittent therapy help improve HBsAg disappearance in chronic hepatitis B patients. Front Microbiol 2022;13:960589, 2022

13.	Lin X, Zheng Y, Li H, Lu J, Ren S, Liu Y, Wang X, Zheng S, Ma L, Cao Z, Chen X. Serum hepatitis B virus large and medium surface proteins as novel tools for predicting HBsAg clearance. Front Immunol 2022;13:1028921, 2022

14.	Takkenberg RB, Jansen L, De Niet A, Zaaijer HL, Weegink CJ, Terpstra V, Dijkgraaf MGW, Molenkamp R, Jansen PLM, Koot M, Rijckborst V, Janssen HLA, Beld MGHM, Reesink HW. Baseline hepatitis B surface antigen (HBsAg) as predictor of sustained HBsAg loss in chronic hepatitis B patients treated with pegylated interferon-α2a and adefovir. Antiviral Therapy 2013;18:895-904, 2013

15.	Thanapirom K, Suksawatamnuay S, Sukeepaisarnjaroen W, Treeprasertsuk S, Tanwandee T, Charatcharoenwitthaya P, Thongsawat S, Leerapun A, Piratvisuth T, Boonsirichan R, Bunchorntavakul C, Pattanasirigool C, Pornthisarn B, Tuntipanichteerakul S, Sripariwuth E, Jeamsripong W, Sanpajit T, Poovorawan Y, Komolmit P. Vitamin D-Binding protein Gene Polymorphism Predicts Pegylated Interferon-Related HBsAg Seroclearance in HBeAg-Negative Thai Chronic Hepatitis B Patients: A Multicentre Study. Asian Pacific journal of cancer prevention : APJCP 2019;20:1257-1264, 2019

16.	Wang WX, Jia R, Gao YY, Liu JY, Luan JQ, Qiao F, Liu LM, Zhang XN, Wang FS, Fu J. Quantitative anti-HBc combined with quantitative HBsAg can predict HBsAg clearance in sequential combination therapy with PEG-IFN-α in NA-suppressed chronic hepatitis B patients. Front Immunol 2022;13:894410, 2022

17.	Wu F, Lu R, Liu Y, Wang Y, Tian Y, Li Y, Li M, Wang W, Zhang X, Jia X, Dang S. Efficacy and safety of peginterferon alpha monotherapy in Chinese inactive chronic hepatitis B virus carriers. Liver International 2021;41:2032-2045, 2021

18.	Wu FP, Yang Y, Li M, Liu YX, Li YP, Wang WJ, Shi JJ, Zhang X, Jia XL, Dang SS. Add-on pegylated interferon augments hepatitis B surface antigen clearance vs continuous nucleos(t)ide analog monotherapy in Chinese patients with chronic hepatitis B and hepatitis B surface antigen <= 1500 IU/mL: An observational study. World Journal of Gastroenterology 2020;26:1525-1539, Apr, 2020

19.	Yan L, Zhu CW, Li J, Chen LW, Ding YZ, Cao ZJ, Liu KH, Lin LY, Tang WL, Xie Q, Xu YM, Bao SS, Wang H. Entecavir add-on or switch-to pegylated interferon improves HBsAg clearance in HBe antigen negative chronic hepatitis B patients. Infection and Drug Resistance 2018;11:2001-2009, 2018

20.	Yang X, Zhang K, Xu Q, Shu X, Mo Z, Xie D, Gao Z, Deng H. Interferon add-on therapy increased clinical cure significantly for interferon-experienced chronic hepatitis B patients with low HBsAg. Front Immunol 2022;13:997608, 2022

21.	Yang Y, Liu M, Deng Y, Guo Y, Zhang X, Xiang D, Jiang L, You Z, Wu Y, Li M, Mao Q. Pretreatment microRNA levels can predict HBsAg clearance in CHB patients treated with pegylated interferon α-2a. Virol J 2018;15:73, Apr 23, 2018

22.	Zhang W, Xing M, Sun W, Chen J, Xie N, Cai Y, Wang Y, Li N, Jiang Y, Zhang F, Wang Y, Zeng Q, Ji Y, Xu C, Jiang C, Song J, Li G. Early Clinical Efficacy of Pegylated Interferon Treatment in Patients with Different Phases of Chronic HBV Infection:A Real-world Analysis. J Viral Hepat 2022, Dec 23, 2022

23.	Zheng CX, Yan HH, Zeng JY, Cai SH, Wu XL. Comparison of pegylated interferon monotherapy and de novo pegylated interferon plus tenofovir combination therapy in patients with chronic hepatitis B. Infection and Drug Resistance 2019;12:845-854, 2019

24.	Zhang M, Li G, Shang J, Pan C, Zhang M, Yin Z, Xie Q, Peng Y, Mao Q, Xiao X, et al. Rapidly decreased HBV RNA predicts responses of pegylated interferons in HBeAg-positive patients: a longitudinal cohort study. Hepatology international 2020;14:212‐224, 2020

25.	Zhang Y, Han J, Zhang X, Li F, Guo Y, He J, Mao R, Zhu H, Yu J, Huang Y, Yang F, Zhang J. Lower frequency of MDSCs was significantly related to functional cure in CHB patients treated with peginterferon. Liver Int 2023;43:329-339, Feb, 2023

26.	Jia HY, Yu GD, Yu J, Zhang XL, Yang LS, Wang B, Zhang JM, Bai L, Zhang XX, Wang K, Zhao P, Yang DL, Zhao YR, Yu YY, Zhang YM, Gu JQ, Ye CY, Cai H, Lu YF, Xiang DR, Yu L, Lian JS, Hu JH, Zhang SY, Jin CL, Yang YD. Immunomodulatory and Antiviral Therapy Improved Functional Cure Rate in CHB Patients with High HBsAg Level Experienced NA. Journal of Clinical and Translational Hepatology,

27.	Ren P, Li H, Huang Y, Jiang J, Guo S, Cao Z, Zhang C, Zhou T, Gan Q, Zhao S, Chen L, Guo Q, Cai W, Wang H, Hu P, Xie Q. A simple-to-use tool for predicting response to peginterferon in HBV DNA suppressed chronic hepatitis B patients in China. Antiviral Res 2021;194:105163, Oct, 2021
